# Colorectal cancer risk: stereotypical assumptions and competing values – a qualitative study with the general public

**DOI:** 10.1186/s12889-026-26737-2

**Published:** 2026-02-19

**Authors:** Erica Sundell, Mariann Hedström, Jessica Nihlén Fahlquist, Jennifer Viberg Johansson, Åsa Grauman

**Affiliations:** 1 Centre for Research Ethics and Bioethics, Uppsala University, Box 564, Uppsala, SE-751 22 Sweden; 2 Department of Public Health and Caring Sciences, Uppsala University, Box 564, Uppsala, SE-751 22 Sweden

**Keywords:** Colorectal, Bowel, Cancer, Risk, Perception, Communication, Lifestyle, Qualitative, General public

## Abstract

**Background:**

Informing the public about lifestyle-related risk factors for colorectal cancer (CRC) is central for cancer prevention. More knowledge is needed about the public’s perceptions of CRC risk and how it relates to their lifestyle decisions, in order to design and communicate risk information effectively. This study aims to explore how the general public perceives risk factors for CRC, their risk of developing CRC, and their willingness to make lifestyle changes to reduce their CRC risk. The study also explores their experiences of, and preferences for, lifestyle-related risk communication and cancer prevention in the community.

**Methods:**

The study employed an explorative qualitative design. Semi-structured interviews were conducted between May 2024 and January 2025 with 25 individuals from the general public in Sweden, including women and men aged 22 to 80 years. The data were analysed using reflexive thematic analysis as described by Braun and Clarke.

**Results:**

Four themes with 10 sub-themes were identified. The first theme, *Information void leaves room for uninformed assumptions*, describes participants’ limited understanding of CRC and its risk factors, resulting in assumptions about risks based on stereotypical and intuitive beliefs. In the second theme, *Colorectal cancer risk – one of many competing aspects in the pursuit of a fulfilling life*, participants described balancing values and factors beyond health risks that influenced their motivation and ability to adopt healthy lifestyle habits. The third theme, *Need for comprehensible information that addresses actual knowledge gaps*, describes that participants requested credible and personally engaging risk information that provides actionable advice without instilling worry or blaming individuals. The fourth theme, *Community interventions should facilitate healthier behaviours but not restrict individuals’ personal choice*, describes participants’ reflections on a shared responsibility for cancer prevention, emphasizing that society should support a healthy lifestyle without excessively interfering in individuals lives.

**Conclusions:**

There was a clear need for more information about CRC and associated lifestyle risks. As individuals balance competing values in their lifestyle decisions, risk information must be nuanced and respectful of personal priorities and communicated carefully in a positive and supportive way to raise awareness and encourage healthy choices.

**Supplementary Information:**

The online version contains supplementary material available at 10.1186/s12889-026-26737-2.

## Introduction

Cancer is a major and growing contributing factor to disease burden and mortality in Sweden and worldwide [1]. Cancer incidence in Europe is rising, and despite a slight decline in mortality rates, estimations indicate a substantial increase in both annual cancer incidence and deaths over the next two decades—unless current risk trends are reversed [2].

Colorectal cancer (CRC) is the fourth most common form of cancer in Sweden, with 7,583 cases in 2021. CRC has multifactorial aetiologies, including heredity and environmental factors, but is also highly linked to behavioural risk factors related to individuals’ lifestyles [3]. Lifestyle-related risk factors were linked to 33–34% of all CRC cases in Sweden in 2018 and include tobacco smoking, overweight or obesity, alcohol consumption, physical inactivity, and dietary habits, i.e., having a high consumption of processed or red meat and a low intake of fruit, vegetables, fibres and calcium [4]. Many Swedes do not meet the national guidelines for physical activity and dietary recommendations [5], and in 2022, 32% of the population were estimated to have a risky consumption of alcohol (ten units of alcohol per week or more) [6]. Furthermore, more than half of the Swedish adult population have overweight or obesity [7].

It is estimated that up to 40% of the cancer burden in the European Union is preventable, which implies a focus on primary and secondary prevention strategies targeting both individuals and the general public [2, 8–10]. Individual preventive action often refers to modifying behaviours to avoid or reduce known harmful exposures. To encourage and inform people on how they can modify their cancer risk, a prevention tool called the European Code Against Cancer (ECAC) has been developed, consisting of 12 recommendations to individuals on how to reduce their risk of cancer. Several of these recommendations relate to the known behavioural risk factors for CRC, including recommendations on avoiding smoking, maintaining a healthy weight, having a healthy diet, limiting alcohol consumption and participating in screening programs [11]. Population-based screening for CRC is expected to be fully implemented in Sweden by 2026 and subsequently offered biennially to individuals aged 60 to 74 years [12]. Awareness of the ECAC in the general public is generally low in several European countries as well as in Sweden [13–15] and the knowledge on cancer risk factors among the general public seem to vary widely both between and within European countries [13, 14, 16–20]. While the awareness of tobacco smoking as a risk factor for cancer is high in several countries, studies indicate that the knowledge of other risk factors including diet, alcohol consumption and overweight is generally modest or low, especially regarding risk factors for specific cancers such as CRC [17–23]. A Swedish study assessing attitudes and knowledge of eligible CRC screening participants found that most perceived diet, heredity and older age as CRC risk factors, while less than half perceived overweight, smoking, alcohol and physical inactivity as CRC risk factors [24]. Awareness of modifiable cancer risk factors has also been linked to sociodemographic factors, where lower awareness levels have been demonstrated among men and individuals with lower socio-economic status or education level [18, 22, 25].

Individuals tend to underestimate the importance of modifiable risk factors in developing cancer [17, 18, 26]. Furthermore, people’s perception of their risk of developing CRC is often inaccurate, and individuals with an elevated risk of CRC commonly underestimate their risk [27]. Raising public awareness about behavioural risk factors for CRC, and providing information on how to reduce the risk, is essential for enabling individuals to make informed decisions about their health. However, a recent study from Sweden found that only 27% of participants reported being likely to make lifestyle changes after learning more about preventive measures. Moreover, very few had previously improved their lifestyle following information on cancer prevention [14]. It is therefore essential to gain a deeper understanding of how individuals in the Swedish general public perceive the risk of CRC and how they view their ability to influence their own cancer risk. It is important to understand which trade-offs individuals face regarding lifestyle choices in relation to reducing CRC risk and how this relates to their willingness to make lifestyle changes. Communicating risk information for cancer prevention is recognized to be complex. Previous studies have indicated that such information risks being misunderstood, perceived as overwhelming, or leading to negative feelings [20, 28–30]. A recent study showed that Swedish individuals with overweight had limited awareness of its link to cancer, and while learning about it felt important and sometimes motivating, it was also emotionally heavy [29]. For CRC patients, lifestyle-related risk information offered hope, yet often came across as moralizing, making them feel judged and personally blamed [30]. To date, no qualitative studies have explored how the Swedish general public perceives risk communication about CRC and lifestyle. In order to design optimal risk information that avoids negative impact, it is essential to learn more about individuals’ experiences and perceptions of risk communication, their attitudes on information efforts in relation to other societal measures, and to get a deeper understanding on their preferences for how the information should be designed and communicated.

This study aimed to explore how the general public perceives risk factors for CRC, their risk of developing CRC, and their willingness to make lifestyle changes to reduce their CRC risk. The study also explored their experiences and preferences for lifestyle-related risk communication and cancer prevention in the community.

## Methods

The study employed an explorative qualitative design.

### Participants

Participants were eligible for inclusion if they were 18 years of age or older and having the ability to read and speak Swedish. The exclusion criterion was a history of cancer. Participants were recruited using diverse recruitment strategies. Invitations to participate were initially spread through posts on Facebook, flyers put up at different university campuses and through the network of researchers. The next step was to post an ad in a local daily newspaper. Individuals willing to participate completed a web form indicating their age and sex. Participants meeting the inclusion criteria were purposively and iteratively selected as the sample developed, ensuring representation across age groups and an equal distribution of men and women. Participants were offered a gift certificate worth 150SEK (≈ 13 €) as a token of appreciation. The final sample size (*n* = 25) was guided by the concept of information power. The study had a broad aim, including capturing diverse experiences and perceptions of risk communication from a diverse study population, whereby a larger sample size was needed [31]. Participants’ characteristics are presented in Table 1.

**Table 1:** Participants’ characteristics

	*N*	Mean, range
Total	25	
Sex		
* Female*	13	
* Male*	12	
Age		51, 22–80
20–29	3	
30–39	4	
40–49	7	
50–59	3	
60–69	1	
70–79	6	
80 and older	1	
Born in Sweden (yes)	23	
Educational level (completed)		
* Primary school*	1	
* Secondary school*	5	
* University*,* post-secondary education*	19	
Training as a health professional (yes)	3 (registered nurses)	

### Data collection

The individual interviews were conducted in Swedish between May 2024 and January 2025 by ÅG, using a semi-structured interview guide (see Supplementary File). The questions addressed participants’ knowledge of colorectal cancer (CRC), beliefs about its causes, self-perceived risk, willingness to modify lifestyle to reduce risk, experiences and preferences regarding risk communication, and attitudes towards prevention. If participants did not spontaneously mention known CRC risk factors, the interviewer briefly provided information about risk factors identified in research to enable assessment of participants’ attitudes towards them. Similar questions have previously been used in interviews with cancer patients [30] and individuals with overweight [29]. To ensure that participants felt as comfortable as possible, the interviews were offered either face-to-face or online via Zoom. Twelve interviews were conducted in person (two in participants’ homes and ten within university facilities), and 13 online (video call through Zoom) after participants were informed about the study and signed a consent form. The interviews, which lasted between 15 and 53 min (mean 31 min), were audio-taped and transcribed verbatim. All personal identifiers were removed from the transcripts to ensure anonymity. The study was approved by the Swedish Ethical Review Authority (Dnr 2023-01526-01) before data collection began.

### Data analysis

The data were analysed inductively using thematic analysis according to Braun and Clark, as it provides a flexible yet structured approach. It also recognises the researchers’ subjectivity as a valuable resource in the analysis process ([32, 33]). Therefore, while the analysis focuses on the participants’ subjective experiences captured through their narratives, we acknowledge that both participants’ experiences and the researchers’ interpretations are influenced by their preunderstanding. While the analysis was mainly carried out by ES and ÅG, interpretations were openly discussed with the entire research team throughout the analysis process to strengthen understanding and reflexivity. The research group consisted of researchers with diverse backgrounds and expertise in public health, caring science, and ethics. Furthermore, several of the researchers have extensive experience in qualitative methodology.

The analysis began with actively reading the transcripts. In the next step, data extracts connected to the study aim were labelled with codes that represent interpreted meaning. The codes were then sorted into potential themes. The themes were reviewed and either collapsed or broken down while going back and forth between transcripts, codes, and themes. The final set of themes and sub-themes were labelled with a brief description to capture its essence. While ES organised and performed the main part of the analysis, all researchers read and coded parts of the data and were actively involved in discussing the analysis. Microsoft Excel (version 2108) was used to organise the analysis.

## Results

Four themes with 10 sub-themes were identified. Themes are presented in Table 2.

**Table 2:** Overview of themes

Themes			
1: Information void leaves room for uninformed assumptions	2: Colorectal cancer risk – one of many competing aspects in the pursuit of a fulfilling life	3: Need for comprehensible information that addresses actual knowledge gaps	4: Community interventions should facilitate healthier behaviours but not restrict individuals’ personal choice

Subthemes			
1.1 An overlooked disease that is rarely discussed	2.1 Lifestyle is guided by the balance between well-being and uncertain risk reduction	3.1 Need for enhanced knowledge and actionable advice	4.1 Creating a society that promotes healthy lifestyles
1.2 Limited awareness shaped by stereotypical assumptions and logical reasoning	2.2 Knowledge and intention alone do not enable a healthy lifestyle	3.2 Personal relevance and credibility of the source build engagement and trust	4.2 Health-promoting regulations should not overly interfere with individuals’ lives
1.3 Compensatory risk assessment marked by closeness and emotional–rational contradictions			4.3 Dividing responsibility between society and the individual

### Theme 1. Information void leaves room for uninformed assumptions

Participants described CRC as a disease that is rarely talked about, which contributes to limited awareness of the disease and its risk factors. Perceptions of the typical patient and personal risk tended to be shaped more by intuitive beliefs.

#### An overlooked disease that is rarely discussed

Many participants expressed that they knew very little about CRC and its risk factors. In contrast to other cancers, they did not know anyone diagnosed with the disease, and noted that you don’t hear as much about CRC as other cancers that are more visible in the media. Participants linked this lack of attention to the taboo surrounding the bowel and bowel diseases, and for some, it led to the assumption that CRC is rare. Others believed it to be relatively common, especially among the elderly. A few participants were very well-informed about the disease or had personal experience through affected friends or family members, and had witnessed first-hand how it impacts life.

*”Cancer is a very common disease*,* but you don’t often hear about bowel cancer. You might have heard of someone’s parent getting it later in life. But it’s not like at my age and among my friends*,* where it feels like every other person is diagnosed with breast cancer or cervical cancer. But you very rarely hear about bowel cancer. So*,* I guess it’s less common. And maybe it’s not something people talk about either.” (Participant 15*,* female*,* age range 50–59).*

CRC was widely perceived by participants as dangerous, though prognosis was seen as dependent on early detection. For some, CRC was particularly frightening and perceived as worse than other cancers, referring to painful examinations, premature death, and burdensome treatments with chronic side effects. This fear and worry made one participant actively seek information to ease her anxiety, while another avoided information about the disease.

*” I’m actually not that informed. It’s one of the diseases I worry about the most. Which might be why I don’t want to read too much about it. When I think about rectal cancer and colon cancer*,* I mostly think about metastases and early death. So*,* I don’t know… I’m a bit afraid of the subject.” (Participant 3*,* female*,* age range 40–49).*

For others, CRC was not perceived as particularly frightening due to surgical options and effective treatment that allow many to survive and live a good life afterwards.

#### Limited awareness shaped by stereotypical assumptions and logical reasoning

Although many participants could not recall receiving information on risk factors for CRC, they assumed that unhealthy lifestyle habits increase the risk. They thought the risk could be reduced primarily through a healthy diet, as well as by engaging in physical activity, limiting alcohol intake, and avoiding smoking. Some emphasized that the overall lifestyle has a greater impact than specific habits.

For several participants who were unaware of the link between lifestyle and CRC risk, it was not surprising for them to learn about it during the interview. It was considered “the same old message” they had heard before, related to other diseases. Many also perceived it as logical, particularly regarding diet, since the bowel is directly exposed to what passes through it. However, several participants found it more difficult to understand how physical inactivity, tobacco use, alcohol consumption, and obesity could affect the bowel. For example, smoking was perceived as mainly affecting the lungs, which was described as being “far from the bowel”.

*” It’s hard to connect the intestines specifically to exercise. So that was interesting. I usually think of other parts of the body in relation to physical activity*,* like the lungs*,* breathing capacity*,* and so on. I’ve never thought about it in that way*,* that it could be related to the intestines.” (Participant 10*,* male*,* age range 30–39).*

Few participants mentioned obesity as an independent risk factor, and when learning about it, it was interpreted as a consequence of poor diet and inactivity rather than a risk in itself.

Awareness of threshold levels for when lifestyle habits become a risk was generally low. Although some expressed that there is no safe level of alcohol consumption, many believed that small amounts of alcohol were harmless and that the risk increased mainly with frequent or heavy drinking. Some guessed that the recommended limits from the government defined the threshold, but there was uncertainty about what those limits were. Likewise, beliefs varied regarding what constitutes an unhealthy diet, and some stated that they did not know what defines an unhealthy diet. Many also felt uncertain about the reliability of the risk information they had encountered, as it often changes and can be contradictory.

Participants shared a stereotypical image of persons typically diagnosed with CRC. Many intuitively imagined an older, overweight man who does not exercise, eats unhealthily, and smokes and drinks heavily. Some were aware that this stereotype may not accurately reflect reality.

*”I guess I instinctively picture a middle-aged man. I don’t know*,* especially when it comes to diet*,* I think of middle-aged men as the ones who eat a lot of meat*,* especially red meat and things like salami with lots of salt*,* and maybe don’t exercise as much either.” (Participant 9*,* female*,* age range 20*–*29).*

For several, this image conflicted with encounters of affected individuals who were both younger and had healthy habits. One participant noted that such stereotypes could lead to putting blame on those affected by the disease.

#### Compensatory risk assessment marked by closeness and emotional–rational contradictions

Participants found it difficult to assess their own risk, and felt uncertain whether their present habits were healthy enough, and how previous behaviour influences risk. There was a tendency to use lifestyle compensatory, a healthy lifestyle could compensate for having heredity or health problems that might increase the risk. Some participants did not express any concern about developing CRC, and had an attitude of “if it happens, it happens.” They also reasoned that cancer risk increases with age and that most people will develop some form of cancer if they live long enough. Many participants perceived their risk as low since they considered themselves healthy and had no family history of CRC. Several referred to their own and their relatives’ generally good health, and perceived a higher risk of developing other diseases more common in their family.

*“You should never say never*,* but we don’t have any illnesses in the family*,* apart from dementia when you get old. […] Otherwise*,* we are very healthy. And I feel that I live as well as I can to avoid it. So*,* it’s nothing I go around worrying about.” (Participant 15*,* female*,* age range 50*–*59).*

Considering CRC to be a rare disease also made the risk appear lower compared to other illnesses. One woman, for instance, thought of breast cancer as a greater risk since she thought that it more commonly affects women.

*”Other types of cancer*,* such as uterine cancer or breast cancer*,* feel more specific to me. Skin cancer too*,* we’ve had that in my family*,* so it feels closer. Then there are other diseases*,* definitely things like cardiovascular problems. Those are more common in my family. Those are the kinds of things I tend to think about*,* like*,* okay*,* there are actually people who have had that*,* so I probably have a genetic component too. But since no one has had bowel cancer*,* it’s not something I think about.” (Participant 6*,* female*,* age range 20*–*29).*

Another woman was aware that she had an increased risk of breast cancer and therefore underwent frequent check-ups. Despite that, she was more worried about CRC, which she described as being based more on a feeling than on logical reasoning.

Participants described that risk information prompts reflection on their own lifestyle and what they could do to influence their risk. For some, risk information served as a confirmation that they already had healthy habits. On the other hand, some expressed that risk information could evoke feelings of worry or fear and while this could be motivating lifestyle changes and make individuals more aware of their own health status and potential symptoms, it also risks causing harm to individuals. Some participants had experienced this themselves and described how thoughts of CRC risk were triggered when they experienced symptoms related to the bowel, which led to worry. Reading about affected persons in the media, or when relatives or friends had been diagnosed with cancer, also led to reflection and worry about their own risk and comparisons of risk factors. One woman perceived herself as very similar to her mother and became deeply worried about developing CRC after her mother was diagnosed.

*” It’s something I’ve started thinking about quite a lot since my mum had problems. She hasn’t been someone who exercises much*,* and she’s not terribly overweight. She and I are quite similar*,* so it makes you a bit worried. So*,* I try to eat vegetables and exercise. I think about it much more than I do about getting breast cancer or something else*,* because that seems more distant. But when it comes to the intestines*,* that’s probably the type of cancer I think I’m most at risk of getting.” (Participant 4*,* female*,* age range 40*–*49).*

Consequently, participants stressed the importance of careful consideration before communicating risk-related information.

### Theme 2: Colorectal cancer risk – one of many competing aspects in the pursuit of a fulfilling life

Participants described a balancing of different values in life that influenced their lifestyle habits. They also expressed that various factors affect their ability and motivation to make lifestyle changes, both in relation to general health risks and specifically to the risk of CRC.

#### Lifestyle is guided by the balance between well-being and uncertain risk reduction

While reduced cancer risk motivated some participants, others found the reduced risk of cardiovascular disease or improvement in existing health conditions to be stronger motivators for maintaining healthy habits or changing unhealthy behaviours. Several participants stated that they did not aim to prevent specific diseases, but rather to build resilience and prevent illness in general. Overall, well-being was highlighted by participants as a central motivation for lifestyle habits, reducing the risk of CRC was rather a beneficial side effect.

*”[…] I don’t think that the risk of bowel cancer is really the driving force to try to get better at keeping up with their regular exercise routine. It’s more about general well-being and perhaps*,* above all*,* cardiovascular disease.” (Participant 17*,* male*,* age range 40*–*49).*

Participants spoke about how they generally relate to risk in life, and stated that “life becomes one big risk” if you constantly analyse it. The reduced risk of CRC was perceived as very uncertain, and many pointed out that you can develop CRC regardless of lifestyle. Experiencing that people close to them had suddenly passed away from CRC had led to the insight that the risk cannot be controlled, and they highlighted the importance of enjoying life in the moment. Restrictions were seen as reducing quality of life, and some risks, like eating enjoyable food or having a beer while watching football on TV, were therefore considered acceptable.

*”The ideal would be to just live clean*,* take your daily walks and eat your vegetables. But most people live a different kind of life and may not be willing to give up what they perceive as life’s pleasures. It becomes part of a life analysis or a risk analysis of life. What kind of life do I want to live?” (Participant 17*,* male*,* age range 40*–*49).*

Most participants, however, were open to making small changes—provided there was an evident effect on CRC risk or even a guarantee that they would not develop CRC. Some found it easier to relate to a clearly defined risk factor, similar to how smoking is linked to lung cancer, compared with a range of risk factors that all contribute to the total risk. Several participants also mentioned screening as a way to actively influence the risk.

*”But when it comes to long-term illness*,* I find it so difficult to relate to. It’s easier to relate to training results or body weight. Then hopefully it will correlate*,* so that if I train*,* it might reduce the risk of lots of other things too*,* but I think it’s easier for me to link things to short-term results.” (Participant 23*,* male*,* age range 40*–*49).*

Some participants stated that it would require being informed that they belonged to a high-risk group for CRC, had a family history of the disease, or had already been diagnosed themselves, in order to feel motivated to change their lifestyle habits.

#### Knowledge and intention alone do not enable a healthy lifestyle

Some participants thought that risk information facilitates preventive action, even subconsciously. However, although some expressed both a need and a desire to change their lifestyle habits, they felt that competing values and external factors often complicate such changes. Many mentioned everyday life and work-related factors, such as lack of time, stress, and physical or mental issues.

Participants emphasized that risk information could evoke feelings of guilt and shame towards themselves and their children if they did not live according to recommendations, especially if they currently lack the ability to change the situation. For individuals with limited financial resources, costs were a barrier, especially the high prices of gym memberships and the increasing cost of healthy food. Some, therefore, emphasized the importance of not blaming individuals who have lived in an unhealthy way or developed a disease, as they believed that shame otherwise potentially could hinder change. However, one participant highlighted that feelings of guilt could also be motivating for individuals to change their habits.

*”When I got divorced a few years ago*,* suddenly it wasn’t obvious for me to buy organic meat any more. Before*,* you thought about toxins and such. But if you end up in a situation where you don’t have much money*,* well*,* then you look for discounts*,* and then you feel bad and stressed and have a bunch of small children… then you buy ready-made food. And if you then receive information telling you that you’ve made really bad choices*,* both for yourself and your children*,* how does that make you feel?“ (Participant 4*,* female*,* age range 40*–*49).*

Participants expressed that norms and social interactions influenced their habits, particularly their eating habits and alcohol consumption. Some noted that alcohol consumption is associated with student life and something that naturally decreases when one starts working and forms a family. However, participants also expressed that it can be difficult to abstain from alcohol because it is expected that socializing involves having a beer or a glass of wine.

Family was perceived to have a significant impact on participants’ dietary habits. For instance, making unhealthy adaptations to suit their children’s preferences or eating healthier thanks to their partner’s initiative. Participants also noted that dietary habits are shaped during childhood and can be difficult to change; some felt that they needed to learn how to prepare new, healthy types of food. Environmental aspects were another factor considered by participants, which could help motivate healthy changes when they agree with risk-reducing actions for CRC, such as reducing red meat consumption. However, participants also stressed that different values could sometimes be in conflict, such as environmental and health considerations regarding fish consumption. Participants described making trade-offs between which values were most important to them, and noted that these priorities could shift during different phases of life.

*“When it comes to food*,* there are many different things to consider. Firstly*,* it should be tasty and contribute to quality of life. […] Right now*,* I let my wallet lead my choices. That is where I am in my life at the moment. When I was younger*,* I was not as frugal.” (Participant 1*,* male*,* age range 20*–*29).*

### Theme 3: Need for comprehensible information that addresses actual knowledge gaps

Participants expressed a need for more understandable information and deeper knowledge about CRC and its links to lifestyle. They highlighted several aspects of how risk information should be communicated and shared their preferences regarding its content.

#### Need for enhanced knowledge and actionable advice

Many participants described being curious and positively inclined toward risk information, and wanted more information about CRC and associated risk factors. They expressed a need for a deeper understanding of the mechanisms behind why something constitutes a risk, stating that the concept of risk is complex and difficult to comprehend. Some emphasized that it is essential to be able to place risk in context and to compare different risks in order to understand which factors have the greatest impact when making lifestyle decisions.

*”But in order to have knowledge about my own body*,* it feels important to me to know roughly what happens on a basic level; it doesn’t have to be super advanced.” (Participant 9*,* female*,* age range 20*–*29).*

Several participants expressed that risk information should be framed in a positive way rather than using fear-based warnings about dangers. Prohibitions and moralizing messages were generally viewed negatively. However, some argued that using warning images or labelling products with warning texts might be necessary for people to understand how the bowel is affected by, for example, alcohol, since such an association can otherwise be difficult to recognize. Participants also noted that it is not helpful to only inform about the risk itself without offering guidance on how to reduce it. Many described that it is better to inform about protective factors and encourage simple ways to reduce risk, rather than focusing on what should be avoided.

*”If you turn it around a bit*,* so that it doesn’t come across as lecturing*,* avoid this and that without saying more… I mean*,* it’s nicer to just hear that if you don’t smoke*,* you’re protecting yourself against cancer*,* compared to saying that if you smoke*,* there’s a risk. So it’s more about what you can do yourself in a positive way.” (Participant 25*,* female*,* age range 30*–*39).*

Some participants thought that it is better to encourage habits that protect against many diseases, rather than linking them to specific illnesses. They suggested talking about gut health and informing about what is generally good for the gut. Participants also requested information with concrete, manageable advice, for example, which foods are good or bad. Participants wanted clear information about threshold values for when something becomes a risk, they noted that words like “avoid” are difficult to interpret and questioned whether it means “never” or “sometimes”. Some participants also highlighted the importance of understanding the potential consequences of CRC and its treatment (such as the use of a stoma), suggesting that this knowledge could motivate people to change their habits to reduce the risk of illness.

#### Personal relevance and credibility of the source build engagement and trust

Participants with prior health issues expressed that it made them more interested in seeking out and engaging with risk information, making it feel more personally relevant, while some participants who perceived themselves as healthy and not belonging to a high-risk group for CRC, did not consider risk information relevant to them.

Participants highlighted that credibility is crucial for trusting and accepting risk information. Many said they trust information from authorities, researchers, and the healthcare system, while tabloid media were viewed as sensationalist and profit-driven, and thus perceived as exaggerated and less trustworthy. Some participants were also sceptical about searching for information online on their own, referring to concerns about encountering unreliable sources. Several participants felt they lacked the expertise to interpret scientific findings on risk, and instead relied on those who communicated the information to have made that assessment.

*“I’m not a researcher*,* so I can’t really assess research reports. But you have to trust things that have been reviewed. […] They often say in these kinds of campaigns that it’s clinically proven. And what does that mean? Then you’d have to look into that*,* and I’m not qualified to read those kinds of reports. You have to trust researchers at universities or district doctors*,* specialists—the people who are really involved in it.” (Participant 13*,* male*,* age range 70–79).*

There were considerable variations in where participants currently seek risk information and where they would prefer to receive it, such as through health-care providers, TV and radio, social media, or newspapers. However, timing and context were considered important, and risk information should be provided when individuals are receptive, for instance, during screening. Some stated they would prefer to receive the information from a doctor with whom they had an established relationship. Some participants expressed that an overload of risk-related information can leave individuals feeling overwhelmed and unmotivated to engage with the content. Therefore, they suggested that risk information should prioritize and only warn about certain risks with the highest impact.

*“You become risk-averse in a way; you can’t cope with all these risks. […] The best thing for me personally… I find it easiest to take things on board and make a change*,* not by becoming afraid or scared*,* but by getting information: there’s an easy way to manage this. If you get the feeling that you’re making a profit when you do something small*,* it becomes fun*,* and then you think*,* yes*,* that’s great*,* I can add another point to my good conscience box.” (Participant 2*,* female*,* age range 40*–*49).*

### Theme 4: Community interventions should facilitate healthier behaviours but not restrict individuals’ personal choice

Participants reflected on the shared responsibility of society and individuals in preventing disease and promoting healthy lifestyles. They shared different perspectives and attitudes toward community interventions and discussed various aspects of public health measures.

#### Creating a society that promotes healthy lifestyles

Many participants thought that the public authorities should, to a large extent, actively work to improve the population’s lifestyle habits. Some emphasized that society needs to take a long-term approach and influence norms in the same way as has been done with smoking, which necessitates continuous information efforts over time. One participant stressed that authorities also need to consider the bigger picture in society; if meat consumption is to decrease, for example, there must also be support in place for farmers to transition the production.

Participants described that society can promote and support healthy habits in several ways, including shaping the community in a way that encourages healthy habits, with interventions starting early in life.

*”More opportunities for exercise in everyday life. I would like to see more investment in public health. […] Right from the start at school*,* I wanted there to be more opportunities for activities and sports in connection with school. More outdoor gyms*,* for example.” (Participant 14*,* female*,* age range 40*–*49).*

Another societal measure that was highlighted was providing support and guidance to individuals who wish to change their habits, for example, through health consultations or clear food labelling that facilitates informed choices in stores. Several participants highlighted a sedentary lifestyle as a major societal issue, noting that society could facilitate greater physical activity and everyday movement. Some examples mentioned included subsidies for gym memberships and activities, the possibility of borrowing exercise equipment or trying out sports, and more bike lanes and outdoor gyms.

#### Health-promoting regulations should not overly interfere with individuals’ lives

Many participants were opposed to bans and regulations that overly interfere with or overly control individuals’ lives. However, several participants expressed ambivalence: while they generally considered total bans and strict regulation to be wrong, believing that individuals must be free to make their own choices, they also acknowledged that a certain level of societal guidance could help people make healthier choices, thereby improving public health over time.

*”I am in favour of doing so to a certain extent. […] There are limits to what can be done. I don’t think alcohol should be banned*,* even though we know that some people drink too much. […] If it turns into paternalism*,* then I am against it. That’s when you start banning things and imposing penalties*,* taxing things and so on.” (Participant 11*,* male*,* age range 30*–*39).*

Some believed that providing information and recommendations is a better way to influence people’s habits than regulation, leaving the decision to the individual. Some participants stated that prohibitions must be supported by strong scientific evidence, noting that there is currently too much uncertainty.

Some participants were positive toward regulations and taxes on unhealthy foods, as well as reduced prices for healthy foods like vegetables, since this makes it easier for individuals to make healthy choices. One participant stated that society must do more through legislation to address advertising and harmful substances.

*”You can ban certain substances that are directly harmful. You can ban alcohol advertising. […] I think society could do much more and has a great responsibility in this area. People all have different possibilities. Some don’t have the same resilience or the same ability to avoid these harmful patterns. There are such powerful economic interests: the alcohol industry*,* the tobacco industry*,* and the red meat industry. […] So there really needs to be some kind of strong counterforce from society.” (Participant 1*,* male*,* age range 20*–*29).*

#### Dividing responsibility between society and the individual

Participants expressed differing views on the responsibility of society and the individuals in preventing disease. Some believed that individuals are responsible for their own health and free to make choices regarding their lifestyle. However, it was stressed that people’s conditions and opportunities to influence their lives differ. Other participants stated that society and individuals share responsibility. One participant described it by saying that costs related to food or exercise are determined at the societal level, but individuals can still make choices such as walking instead of driving.

*”I think people have a great deal of control over their lives. So*,* of course*,* at the societal level*,* we need to ensure that everyone has the opportunity to make healthy decisions. But if people don’t make those choices*,* maybe it becomes more their own… you still have to take responsibility for what you’ve done and the decisions you’ve made. At the same time*,* it’s very difficult to blame anyone*,* to say that it’s because you did this that you got cancer. That feels a bit lacking in empathy.” (Participant 6*,* female*,* age range 20*–*29).*

Some participants believed that the authorities are responsible for disease prevention and public health. They should protect citizens from illness and are responsible for providing information and making decisions based on scientific evidence, even when those decisions are unpopular.

Participants also emphasized that society needs to work with prevention at all levels and support individuals who lack the knowledge or financial resources. They thought that information must reach the entire population so that knowledge about health and lifestyle does not become a class issue.

## Discussion

Overall, participants expressed that they knew very little about CRC, and most had not encountered specific information about associated risk factors. CRC was perceived as a disease that is not widely discussed in society. Hence, most participants were positive towards receiving more information about the disease and its risk factors. They shared perspectives on how the information should be designed and communicated while also highlighting possible negative aspects of communicating about lifestyle risks. Many felt that society plays an important role in disease prevention, but also emphasized that it is up to individuals to make choices about their lifestyle and that there are many other important values in life beyond disease prevention.

In our study, participants’ awareness of lifestyle factors associated with CRC were characterized by considerable ambiguity. This is consistent with previous research, demonstrating that public awareness of risk factors associated with specific types of cancer is limited in several European countries [17–23]. Consequently, many based their understanding of risk factors on assumptions and logical reasoning, and therefore struggled to comprehend how lifestyle factors not directly linked to bowel function could influence the risk. Individuals tend to underestimate the impact of lifestyle factors beyond the classic risks for cancer such as smoking, for example risks relating to specific foods, obesity or alcohol consumption [17, 18, 26]. This may lead individuals to overestimate the healthiness of their own lifestyle and, consequently, underestimate their personal risk. This is supported by previous research demonstrating that individuals often misperceive their risk of developing CRC [27, 34], and those at higher risk commonly underestimate their risk [27]. While participants in our study generally acknowledged lifestyle as an important factor influencing CRC risk, they were uncertain about how specific behaviours and threshold levels relate to that risk. For instance, it was widely assumed that the risk of alcohol increases mainly with frequent or heavy drinking, and that small amounts are harmless. Limited awareness of how alcohol consumption relates to cancer risk has previously been reported in relation to breast cancer, particularly among individuals who consume alcohol [35].

This illustrates that the concept of risk can be challenging to comprehend, both in terms of general understanding and in relation to one’s own behaviour. Difficulties in estimating personal CRC risk have been demonstrated in a previous study, which identified differences in risk perception between CRC screening participants and non-participants, with a higher proportion of non-participants reporting being unaware of their CRC risk [24]. Several participants argued that absolute risk offers a better understanding of how lifestyle factors relate to the overall risk for CRC. Communicating risk in absolute rather than relative terms, and clarifying how it changes from pre-existing baseline levels, has previously been suggested as preferable, since it provides a more accurate representation of both the risk itself and the potential impact of interventions [36]. Participants tended to assess their own risk of developing CRC as low or moderate, referring to their generally healthy lifestyle and the absence of a known family history of CRC. However, many also felt uncertain whether their lifestyle was sufficiently healthy, and requested a deeper understanding of risk factors to compare and contextualize how the risk is influenced. This indicates that, although many cancer types share common risk factors, risk communication should avoid be overly general but instead clearly address lifestyle‑related risk factors specific to CRC.

Many participants noted that other cancer diagnoses are more visible in society and the media than CRC. While some stated that knowing someone affected by CRC or reading about it had triggered thoughts about their own risk, many perceived CRC as a rare disease, typically affecting older men with unhealthy lifestyles. Several female participants stated that they felt more likely to develop other cancers, such as breast cancer. In contrast to CRC, breast cancer is generally seen as more common and as affecting younger women—a perception largely shaped by media portrayals [37]. This reflects an important aspect of risk perception, referred to as heuristic processes, which involve assessing the likelihood of developing a disease based on how easily examples come to mind (e.g., knowing someone affected or reading about it in the media) and on perceived similarity to those who develop the disease [37]. This indicates that it is important to make CRC more visible in society and to provide a more nuanced image of the disease, both visually and in content, to avoid reinforcing stereotypes that contribute to inaccurate perceptions of susceptibility. Such misperceptions may make younger individuals less likely to notice or act on early symptoms because they do not identify with the stereotypical CRC patient.

The aim of informing individuals about lifestyle-related risk factors for CRC is to raise awareness and facilitate informed decision-making. Participants emphasised that risk information needs to be perceived as personally relevant, in order to be engaging. Some stated that having prior health issues made them more interested in risk information for CRC, while others considered themselves as healthy and not part of a high-risk group and therefore did not perceive it as relevant. Communicating tailored risk information, based on personal risk factors for CRC, has been shown to increase individuals’ perceived susceptibility and the perceived relevance of the information [38]. However, awareness of risks does not necessarily motivate individuals to make lifestyle changes [13, 14]. Most participants did not consider the reduced risk of CRC as a central motivation for maintaining a healthy lifestyle or changing unhealthy behaviours. Instead, lifestyle choices were primarily guided by factors that promote general health and well-being. Some risks were considered acceptable if it meant enjoying life here and now, particularly when the benefits of change appeared vague or uncertain. This suggests that risk communication that considers individuals’ priorities and well-being, while also being specifically tailored to their personal risk, may be perceived as more relevant and motivating.

Although participants were generally positive towards receiving more information about lifestyle risks and CRC, they also raised concerns. Participants argued that an overload of risk-related information could lead individuals to feel overwhelmed and unmotivated to engage with the content, as demonstrated in previous studies [20, 28]. Some had experienced that it made them more aware of possible symptoms and concerned about their own risk, which led to fear and worry. Participants suggested that risk communication should prioritize the most important risks. This aspect has previously been raised by individuals with overweight or obesity, with the argument of avoiding reinforcing the feeling that “everything causes cancer” [29]. Participants also stressed that risk information about lifestyle could place blame or guilt on individuals with risky lifestyle habits or be stigmatizing for those affected by CRC. They argued that the information should have a positive tone, highlighting protective factors and manageable changes rather than fear-based messaging focusing on risks. Raising awareness about cancer by communicating in a positive and hopeful manner has previously been suggested by patients with breast cancer and CRC as a way to reduce stigmatization [30]. Furthermore, participants stressed that lifestyle-related risk information should be communicated in a suitable context when individuals are receptive to it, for instance, in connection with screening together with the invitation- or results letter, or at a health check-up, preferably coming from their general practitioner. This is supported by a previous finding, demonstrating that individuals were generally open to receiving lifestyle-related advice during a screening visit [39]. Whether and how CRC screening can function as an effective information opportunity therefore warrants further investigation. Multiple, parallel information strategies are likely required. Even when individuals have both knowledge and motivation to change lifestyle habits, they face barriers that could complicate such efforts. Many participants expressed everyday life challenges that influenced their lifestyle, as well as the impact of their social environment. Successful cancer prevention requires a dual approach that supports individual efforts by governmental policies and actions [11]. Participants argued that individuals and society have a shared responsibility to prevent disease and that societal measures should offer support and guidance. Support strategies aimed at individual and societal levels could include, e.g., clear food labelling and regulations on tobacco and alcohol [20]. Interestingly, several participants expressed ambivalence about identifying themselves as liberal and being opposed to excessive societal interference, while also acknowledging that certain measures, such as bans or taxes, could be necessary to promote healthier choices. This suggests that health is regarded as a highly valued priority that could make some restrictions on personal freedom of choice acceptable.

### Strengths and limitations

To our knowledge, this is the first study to explore how individuals in the Swedish general public understand risk factors for CRC and how this relates to their perceived ability and motivation to influence their personal risk as well as their preferences for risk communication about CRC. The results provide valuable insights that can guide future research and improve strategies for CRC prevention and risk communication.

Using an inductive approach, we sought to explore the research questions in depth and provide a rich description of the dataset. The study’s broad scope and heterogeneous sample generated extensive material, potentially limiting the depth of analysis. Nevertheless, the analysis resulted in a comprehensive description of a topic where knowledge is currently limited. Furthermore, the inclusion of multiple researchers in the analysis process can both enhance reflexivity and add depth to the interpretation of the results [33].

We employed various recruitment strategies to reach and include participants with different backgrounds. The sample was diverse in terms of sex, educational level and age, but had an overrepresentation of highly educated individuals. Given that the study aimed to represent the general public, this introduces a potential bias, which may limit the transferability of the findings [40]. Furthermore, the majority of participants were born in Sweden which also represents a limitation of the study as the results might not reflect views of sub groups in society. Cultural and sociodemographic factors influence awareness of lifestyle-related cancer risk factors and attitudes regarding lifestyle, for instance, in relation to dietary practices [18, 22, 25]. Future research should aim to include individuals from diverse cultural backgrounds to explore potential differences in understanding of CRC risk factors and preferences for risk communication. Participants received a gift card as a token of appreciation for their participation in the study. We do not believe this influenced their decision to participate, as the amount was modest and many participants expressed that their participation was driven by an interest in research and the topic itself.

## Conclusion

The results of this study suggest that CRC needs to be made more visible in society in order to raise awareness and reduce preconceived assumptions about the disease. We identified an unmet need to understand how specific lifestyle habits relate to risk for CRC. We can conclude that risk information must be clear and personally engaging to be effective, but should be communicated with care, without exaggeration or fear-based messaging. Risk communicators must recognize that individuals have their own perceptions of what contributes to their well-being and make personal trade-offs between quality of life and risk reduction. This suggests that risk communication about CRC should be nuanced and respectful of individual values and priorities. While society should promote healthy choices, to be both ethically legitimate and effective, the information must avoid becoming paternalistic or judgmental toward those who do not follow expert recommendations, especially given that such recommendations can never guarantee protection against cancer.

## Supplementary Information

Supplementary Material 1.

## Data Availability

Due to the formulation of the informed consent form, study data cannot be made publicly available. Data are, however, available from the corresponding author upon reasonable request subject to ethical permissions and participant consent.

## Abbreviations

CRC: Colorectal cancer
ECAC: European Code Against Cancer
RTA: Reflective thematic analysis
