# Supplementary material for: Colorectal cancer risk: stereotypical assumptions and competing values – a qualitative study with the general public
Source: BMC Public Health. 2026 Feb 19;26:706. doi: 10.1186/s12889-026-26737-2 (PMC12930923; doi:10.1186/s12889-026-26737-2)
Supplement: Supplementary file 1 — Supplementary Material 1. [file 12889_2026_26737_MOESM1_ESM.docx]

**Supplementary file. Interview guide. AI-assisted translation from Swedish.**

**Opening Questions**

- What do you already know about bowel cancer colon and rectal cancer (bowel cancer)? Is it something you have heard about before?
- Do you have any perception of how dangerous or serious the disease is?
- Do you have any idea of how common it is?
- Do you have any perception of who gets bowel cancer? How do you picture the typical patient?

**Main Questions**

**Risk Factors**

- Cancer can be caused by many different factors. What are your thoughts on what causes bowel cancer, or what increases the risk of developing it? *Can you think of any other causes or risk factors?*
- If you were to think instead about things that protect against bowel cancer, what might they be? *Can you think of several?*
- To what extent do you think it is possible to influence your own risk of developing bowel cancer?
- (If not mentioned by participants: to what extent do you think exercise/alcohol/smoking/dietary habits affect the risk of colon and rectal cancer?)
- When it comes to dietary habits, are there any particular foods you think are especially risky or protective?
- You have mentioned several possible risk factors (such as …), which do you think has the greatest impact on risk?
- How much alcohol do you think one needs to drink before it becomes a risk?
- How much exercise is needed to provide protection?
- How important do you think lifestyle habits are compared to, for example, heredity, chance, or other exposures?
- What do you think influences the risk the most?

**Self-Perceived Risk**

- How do you view your own risk of developing bowel cancer in the future? (For example, is it lower, the same, or higher than other people’s risk?) Which risk factor is most important for you to consider? Is it something you worry about?
- What do you base that perception on?

**Willingness to Change Lifestyle to Reduce Risk**

- Would you consider changing anything in your daily life to reduce your risk of bowel cancer? For example, exercising more, reducing alcohol consumption, quitting tobacco, losing weight, changing your eating habits?
- Is there any lifestyle habit that feels easier or harder to change?
- Are there changes that seem more difficult than others? Or changes you would not want to make?
- Is there anything that would make it easier for you to maintain healthy lifestyle habits? Is there anything that makes it harder for you to maintain healthy habits?

**Risk Information**

- Can you recall any occasion when you have received information about what increases or decreases the risk of bowel cancer? Can you tell me about that occasion? Where did you get that information? Do you remember how you reacted to it? Did you think anything in particular?
- Could information affect you negatively in any way? Could it be perceived as offensive?
- Are you personally interested in receiving information about cancer risk factors? In what way is it useful? What is important or interesting for you to know?
- How would you like information about lifestyle and cancer risk to be communicated?
- Who should communicate it?
- Are there occasions that are better suited for such information?
- Are there occasions that are less suitable for such information?

**Society’s Responsibility to Prevent Cancer**

- To what extent do you think authorities should work to prevent cancer?
- Do you have any thoughts on cancer should be prevented?
- To what extent do you think authorities should work to improve the population’s lifestyle habits in order to prevent cancer cases in the long term? Do you have any thoughts on how people’s lifestyle habits could be improved?

**Closing Questions**

That was all the questions I had. [Give a short summary of what has been said]. Is there anything you would like to clarify? Is there anything we haven’t talked about that you would like to add?

Thank you for your participation!
